# Nutritional and Biochemical Assessment of Edible Fruits From Angola's Native Flora

**DOI:** 10.1002/fsn3.70283

**Published:** 2025-06-01

**Authors:** Josefa Rangel, Ângela Liberal, Tânia C. S. P. Pires, Tiane C. Finimundy, Lillian Barros, Filipa Monteiro, Maria M. Romeiras, Ângela Fernandes

**Affiliations:** ^1^ Linking Landscape, Environment, Agriculture and Food Research Center (LEAF), associated Laboratory TERRA Instituto Superior de Agronomia (ISA), Universidade de Lisboa Lisboa Portugal; ^2^ Centro de Botânica, Universidade Agostinho Neto Luanda Angola; ^3^ CIMO, LA SusTEC, Instituto Politécnico de Bragança Bragança Portugal; ^4^ Centre for Ecology Evolution and Environmental Changes (cE3c) & CHANGE ‐ Global Change and Sustainability Institute, Faculdade de Ciências, Universidade de Lisboa Lisboa Portugal

**Keywords:** African edible fruits, antimicrobial, antioxidant, phenolic compounds, phytochemical profile

## Abstract

Angola's native flora hides a wealth of underexplored edible fruits with significant nutritional and bioactive potential. This study provides a comprehensive evaluation of the physicochemical and bioactive potential of some less explored edible fruits from this country, namely *Annona muricata* , *A. squamosa* , *A. senegalensis* , *A. boehmii*, *Dacryodes edulis* , and *Strychnos spinosa* . Nutritional profiling revealed carbohydrates as the predominant macronutrient, with *A. muricata* exhibiting the highest concentration (93.3 g/100 g dw). *D. edulis* , in turn, stood out for its high fat content (40.5 g/100 g dw), while *A. senegalensis* contained the highest protein levels (15–6 g/100 g dw). Free sugars analysis highlighted *A. squamosa* for its elevated fructose and glucose levels, contributing to its pronounced characteristic sweetness, whereas *D. edulis* exhibited minimal sugar content (1.27 g/100 g dw). Organic acid profiling revealed malic acid as dominant. Saturated fatty acids were predominant across most fruits, with *A. boehmii* showing the highest levels (71.6%), while *A. senegalensis* was enriched in monounsaturated fatty acids (45.15%). The phenolic analysis revealed a rich profile in bioactive compounds, with *A. muricata* and *A. squamosa* exhibiting significant levels of quercetin‐3‐*O*‐rutinoside, while *A. boehmii*, *S. spinosa* , and *D. edulis* were characterized by distinct major phenolic compounds, such as cyanidin‐3‐*O*‐glucoside, isorhamnetin‐3‐*O*‐glucoside, and corilagin, respectively. Antioxidant activity was strongest in *D. edulis* and *A. senegalensis* (EC_50_ = 0.18 and 0.25 μg/mL, respectively), while *A. squamosa* exhibited notable antibacterial activity (MIC = 0.3 mg/mL). This study underscores the nutritional and bioactive potential of Angola's native fruits, highlighting their applications in the food, nutraceutical, and pharmaceutical industries.

## Graphical abstract

This study provides a comprehensive evaluation of the physicochemical and bioactive potential of underexplored edible fruits of *Annona muricata* , *A. squamosa* , *A. senegalensis* , *A. boehmii*, *Dacryodes edulis* , and *Strychnos spinosa* from Angola. Macronutrient composition (e.g., carbohydrates, fats, proteins), phenolic compounds, antioxidant activity, and antibacterial properties were evaluated. This study underscores the nutritional and bioactive potential of Angola's native fruits, highlighting their applications in the food, nutraceutical, and pharmaceutical industries.

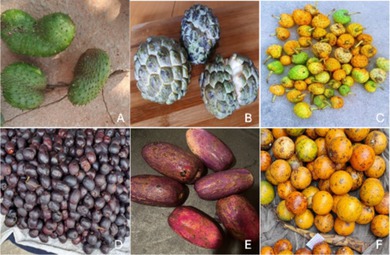

## 1 Introduction

In developing countries, global population growth, deforestation, and climate change are major drivers of food and nutrition insecurity, primarily stemming from inadequate access to sufficient and nutritious food (Narjes and Lippert 2019). In the African continent, particularly Angola, despite the nation's relatively high *per capita* income, these problems endure, with a significant portion of the population experiencing poverty and malnutrition (Baumgärtel et al. 2023). In this scenario, native edible fruits represent a promising solution to nutritional challenges, as they are well adapted to local environments, deliver essential nutrients, and play a significant role in improving food security (N'Danikou et al. 2017; Yasin et al. 2018).

Though Angola is rich in diverse wild edible fruits, their contribution to daily nutrition remains limited, as these are consumed sporadically and frequently stared at as low‐quality “famine foods” (Baumgärtel et al. 2022). Over the years, *Annona* species have earned significant notice for their phytochemical and bioactive potential (Rangel et al. 2024). *Annona muricata* Linn is a tree fruit widely distributed throughout the tropical and subtropical regions of the world, including Africa (Zubaidi et al. 2023). Commonly known as soursop or graviola owing to the fruit's sweet and sour flavor, *A. muricata* is one of the most widely used medicinal plants due to its richness in bioactive compounds. The fruit pulp is consumed and used as an ingredient in various foods and beverages (Patel and Patel 2016), being appreciated for its flavor and rich profile in essential minerals and vitamins C and E (Coria‐Téllez et al. 2017; Marquez et al. 2012; Pareek et al. 2011). Similarly, *Annona squamosa* , traditionally known as custard apple, sweet custard apple, and sugar apple, is a small semi‐branched tree (Kumar et al. 2020) known for its edible fruits and medicinal assets. The fruit pulp is mainly made up of water, proteins, and carbohydrates, along with essential minerals, vitamins, and a small amount of lipids, making it an essential food in the diet of populations where malnutrition endures (Mainasara et al. 2018). Its rich nutritional profile enables its use as an ingredient in processed foods, enhancing flavor and nutritional value (Kumar et al. 2021). *Annona senegalensis* Pers., a versatile tree species known as the wild custard apple, is also valued for its food and medicinal uses across the African continent, with its fresh fruits representing a valuable dietary component for humans and wildlife. Despite its extensive use in Africa, particularly in traditional medicine, research on the nutritional potential of its edible parts remains limited (Donhouedé et al. 2023).

Other species of edible fruits have been explored on the African continent, not only for their nutritional value but also for their chemical and bioactive properties. For example, Loengo (*Anisophyllea boehmii* Engl.) occurs naturally in African countries in high rainfall areas, such as Angola (Chen et al. 2015; Nkengurutse et al. 2016). *Anisophyllea boehmii* produces an edible stone fruit with a sweet and sour flavor, mainly consumed fresh or processed into juice or jelly. The pulp and peel are the most edible parts, with the stone making up a significant proportion of the fruit (Ibrahim et al. 2015). Little is known about the overall phytochemical and bioactive properties of the *A. boehmii* fruits, limiting their use as food and as a possible source of bioactive compounds. Another example is the evergreen dyonic tree *Dacryodes edulis* , the African pear tree known as safou. *D. edulis* produces an edible fruit rich in nutrients such as lipids, vitamins, and proteins, holding a high content of fixed and essential oils (Ajibesin 2011). The fruits are popular in the diet of many Africans and can be eaten raw, roasted, cooked, and used as a garnish or as a type of butter to eat with bread. The pulp is the only edible part of the fruit, constituting an important source of essential fatty acids (Kadji et al. 2016). The plant is commonly used in traditional medicine in some African countries to treat wounds, skin diseases, dysentery, and fever. Finally, *Strychnos spinosa* Lam, one of southern Africa's most nutritious and prevalent indigenous fruit species, produces nutrient‐dense fruits that provide an essential food source for marginalized rural populations (Kadji et al. 2016). The fruits of *S. spinosa* have a characteristically high concentration of vitamin C, iron, and zinc, with the capacity to improve the diet of these individuals, their nutritional composition being comparable to other traditional fruits, such as strawberries and oranges (Kadji et al. 2016).

While the phytochemical profiles and bioactive properties of various plant parts (roots, stems, leaves, seeds) of these Angolan plant species have been investigated, research specifically focusing on their edible fruits is scarce. Thus, the present study aims to address this knowledge gap by conducting a comprehensive evaluation of the nutritional, chemical, and bioactive profiles of *Annona muricata* , *A. squamosa* , *A. senegalensis* , *Anisophyllea boehmii*, *Dacryodes edulis* , and *Strychnos spinosa* native to Angola, with the ultimate goal of promoting their utilization to enhance nutrition and explore potential medical applications in regions with limited access to these goods.

## 2 Materials and Methods

### 2.1 Samples

**FIGURE 1:**
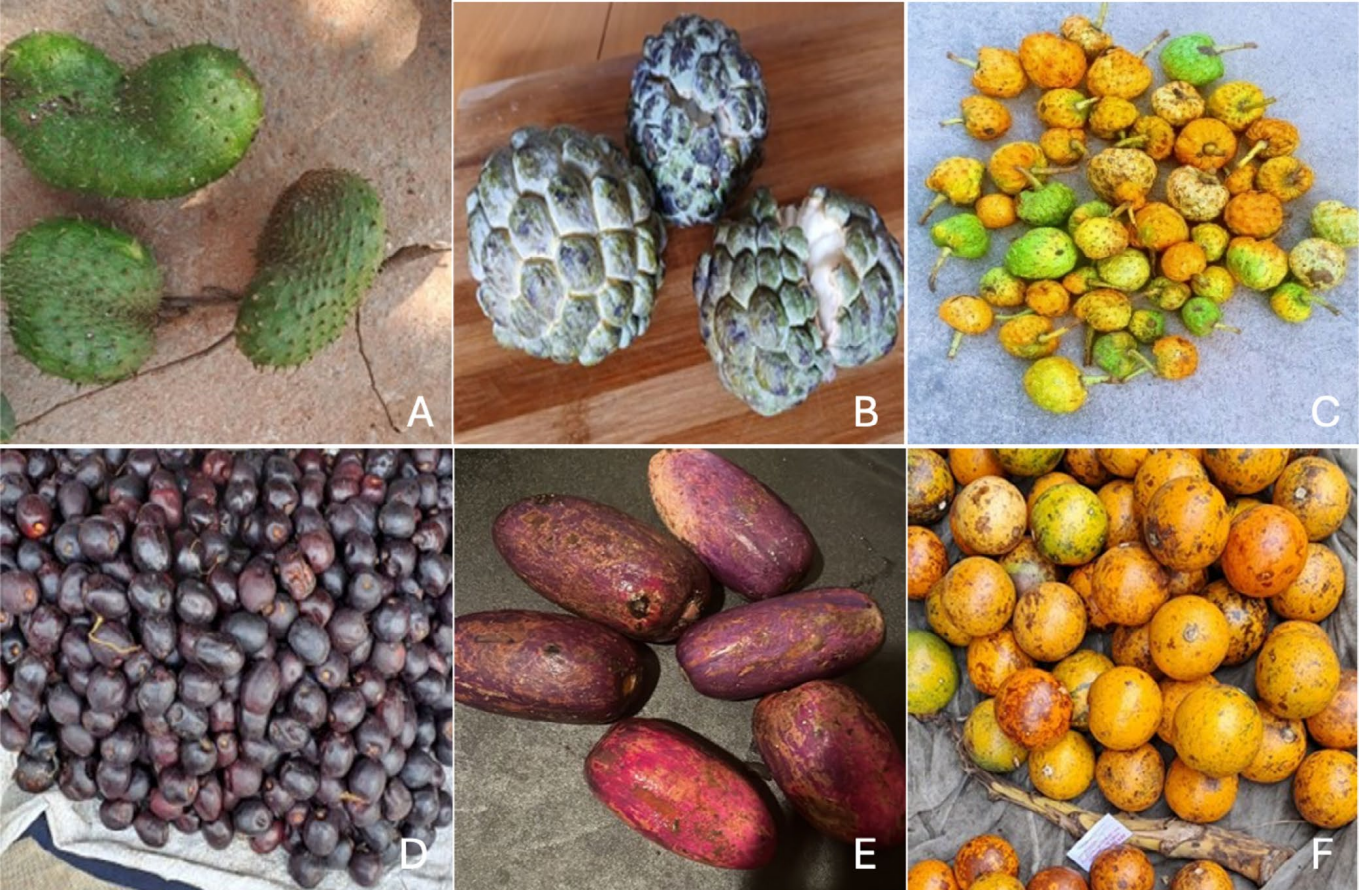
Fruits of *A. muricata* (A), *A. squamosa* (B), *A. senegalensis* (C), *A. boehmii* (D), *P. edulis* (E), and *S. spinosa* (F) (photos by the author, Josefa Rangel).

The edible fruits of *Annona muricata* , *A. squamosa* , *Anisophyllea boehmii*, *Dacryodes edulis* , and *Strychnos spinosa* were collected during the dry season (June–September) in 2022, while *A. senegalensis* was harvested during the rainy season (January) in 2023 (Figure 1). Most samples were collected in Malanje province, Angola, with *A. squamosa* sourced from Cuanza Sul and *Dacryodes edulis* from Uíge provinces. These fruits are commonly harvested by local communities, and small orchards of *A. muricata* and *A. squamosa* are also present.

All samples were cleaned to remove foreign materials, freeze‐dried (FreeZone 4.5, Labconco, MO, USA), and kept in a dry environment until further processing. Freeze‐dried fruit samples were ground in a laboratory‐scale Foss Knifetec 1095 mill and stored in the dark until further analysis.

### 2.2 Extracts Preparation

Hydroethanolic extracts were prepared by suspending 1 g of each lyophilized sample twice in 30 mL of ethanol/water (80:20; *v/v*). The mixture was stirred at 150 rpm for 1 h at room temperature and filtered through Whatman No. 4 paper. The ethanol was removed using a rotary evaporator (Büchi R‐210) at 40°C. The remaining aqueous phase was frozen and lyophilized using a FreeZone 4.5 freeze dryer for subsequent analysis (Bessada et al. 2016).

### 2.3 Chemical Parameters

#### 2.3.1 Nutritional Value

The nutritional composition of *A. muricata* , *A. squamosa* , *A. senegalensis* , *A. boehmii*, *D. edulis* , and *S. spinosa* edible fruits was evaluated using standard AOAC methods (AOAC 2016). In brief, crude protein content was assessed using the macro‐Kjeldahl method (N x 6.25) with an automatic distillation and titration system. Crude fat content was determined through Soxhlet extraction with petroleum ether, and ash content by incineration at 550°C ± 5°C. Total carbohydrates were calculated by difference using the formula: g/100 g dry weight (dw) = *(100 − (g ash + g proteins + g fat))*. The energy content was estimated using the equation: *Energy (kcal/100 g) = 4 × (g proteins + g carbohydrates) + 9 × (g fat)*.

#### 2.3.2 Free Sugars

Free sugars were analyzed using high‐performance liquid chromatography with a refractive index detector (HPLC‐RI; Knauer, Smartline systems), following previously described methods (Spréa et al. 2020). In short, the extraction process involved adding melezitose (5 mg/mL) as an internal standard to 1 g of each dried sample powder, followed by extraction with 40 mL of 80% aqueous ethanol at 80°C for 90 min. After centrifugation, the supernatant was concentrated, defatted with ethyl ether, and further concentrated before being dissolved in water to a final volume of 5 mL and filtered for injection (0.2 μm nylon filters from Whatman) in an HPLC‐RI system. After separation, the free sugar compounds were identified by comparison with standards of their relative retention (Rt), while quantification was made by the IS, with calibration curves constructed with standards. Raw data were processed through the Clarity 2.4 software package (DataApex, Prague, Czech Republic) and expressed in g per 100 g of dw.

#### 2.3.3 Organic Acids

The organic acids profile of the studied Angolan edible fruits was analyzed using ultra‐fast liquid chromatography with a photodiode array detector (UFLC‐PDA; Shimadzu Corporation). The extraction process involved stirring 2 g of each sample with 25 mL of metaphosphoric acid at 25°C and 150 rpm for 25 min, followed by filtration through Whatman No. 4 paper and 0.2 μm nylon filters (Barros, Pereira, and Ferreira 2013). Compound separation was performed on a reverse‐phase 18 SphereClone column (Phenomenex) at 35°C, using a 3.6 mM sulfuric acid solution as the eluent at a 0.8 mL/min flow rate. Compounds were identified by comparing chromatograms with commercial standards, and quantification was done by correlating peak areas at 215 nm with calibration curves. Results were expressed in g/100 g of dw.

#### 2.3.4 Fatty Acids

Fatty acids in the lipid fraction obtained from Soxhlet extraction were characterized after transesterification of the lipid fraction obtained after Soxhlet extraction and were subjected to a methylation process with methanol/sulfuric acid/toluene (2:1:1 (v/v/v); 5 mL) in a water bath (12 h; 50°C; 160 rpm). Deionized water (3 mL) and diethyl ether (3 mL) were added to obtain phase separation and recover the FAME phase (Barros, Pereira, Calhelha, et al. 2013). A YOUNG IN Chromass 6500 Gas Chromatography (GC) System (YL Instruments, Anyang, Korea) equipped with a *split/splitless* injector, a flame ionization detector (FID), and a Zebron‐Fame column (30 m × 0.25 mm × 0.20 μm, Phenomenex, Lisbon, Portugal) was used in the analysis. The elution and operation conditions were previously described by Spréa et al. (2020). The identification and quantification were determined by comparing the relative retention times of the FAME peaks of the samples with commercial standards (FAMEs). The results recorded were processed using CSW 4.0 Software (Informer Technologies Inc., Solihull, UK). The results were expressed in relative percentages of each fatty acid compound.

#### 2.3.5 Tocopherols

Tocopherol content was determined following a procedure previously described by the authors Spréa et al. (2020). Butylhydroxytoluene (BHT; 10 mg/mL; 100 μL) and internal standard (IS; tocol; 50 μg/mL; 400 μL) solutions, prepared with hexane, were added to the lyophilized fruit samples. The extraction procedure consisted of successively adding methanol (4 mL), hexane (4 mL), and saturated sodium chloride aqueous solution (2 mL). The extract was then centrifuged, and the clear upper layer containing tocopherols was collected into a vial, protected from light exposure. The described extraction procedure was repeated twice with hexane, and the combined extracts were dried under a nitrogen stream. To perform the analysis, the dried bell pepper extracts were re‐dissolved in n‐hexane (2 mL), passed through a sodium sulfate anhydrous micro‐column, and filtered (0.2 μm nylon filters from Whatman) into a dark injection vial. An HPLC system coupled to a fluorescence detector (FP‐2020; Jasco, Tokyo, Japan) programmed for excitation at 290 nm and emission at 330 nm was used. The identification was achieved by chromatographic comparison with authentic standards, and the quantification was based on the fluorescence signal response of each standard, using the internal standard (IS; tocol; 50 mg/mL) method and calibration curves constructed with commercial standards. The results were expressed in mg per 100 g of dw.

### 2.4 Phenolic Composition

The phenolic composition of *A. muricata* , *A. squamosa* , *A. senegalensis* , *A. boehmii*, *D. edulis* , and *S. spinosa* edible fruits hydroethanolic extracts was analyzed as previously described by the authors (Bessada et al. 2016). Each extract was redissolved in ethanol: water (80:20, *v/v*) to a final 10 mg/mL concentration and filtered through 0.22 μm disks. Chromatographic analyses were performed in a Dionex Ultimate 3000 HPLC (ThermoScientific, San Jose, California, USA) system equipped with a diode array detector coupled to an electrospray ionization mass detector (LC‐DAD‐ESI/MSn), a quaternary pump, an auto‐sampler (kept at 5°C), a degasser, and an automated thermostatted column compartment. Chromatographic separation was achieved with a Waters Spherisorb S3 ODS‐2C18 (3 μm, 4.6 mm × 150 mm, Waters, Milford, Massachusetts, USA) column thermostatted at 35°C.

The solvents used were: (A) 0.1% formic acid in water, (B) acetonitrile. The elution gradient established was isocratic 15% B (5 min), 15% B to 20% B (5 min), 20%–25% B (10 min), 25%–35% B (10 min), 35%–50% B (10 min), and re‐equilibration of the column, using a flow rate of 0.5 mL/min. The system operated in both positive and negative ionization modes, with detection wavelengths set at 280 nm. MS detection was performed in negative mode, using a Linear Ion Trap LTQ XL mass spectrometer (Thermo Finnigan, San Jose, California, USA) equipped with an ESI source. Nitrogen served as the sheath gas (50 psi); the system was operated with a spray voltage of 5 kV, a source temperature of 325°C, and a capillary voltage of −20 V. The tube lens offset was kept at a voltage of −66 V. The full scan covered the mass range from m/z 100–1500. The collision energy used was 35 (arbitrary units). Data acquisition was carried out with the Xcalibur data system (Thermo Finnigan, San Jose, California, USA).

The phenolic compounds were identified by comparing their retention times, UV‐is and mass spectra with those obtained from standard compounds, when available. Otherwise, compounds were tentatively identified by comparing the obtained information with available data reported in the literature. For quantitative analysis, a calibration curve for each available phenolic standard was constructed based on the UV signal.

Quantification was done using 7‐level calibration curves based on UV signals of available standards or similar compounds when exact standards were unavailable. Results were expressed in mg/g of extract.

### 2.5 Bioactive Properties

#### 2.5.1 Antioxidant Activity

For the antioxidant activity assays, the lyophilized hydroethanolic extracts were dissolved in ethanol: water (80:20, *v/v*) and subjected to successive dilutions from 10 to 0,156 mg/mL.

DPPH radical‐scavenging activity: This methodology was performed using a SpectroStar nano‐spectrophotometer reader (Labtech, Ortenberg, Germany). The reaction mixture in each of the 96 wells consisted of one of the different concentration solutions (30 μL) and methanolic solution (270 μL) containing DPPH radicals (6 × 10^−5^ mol/L). The mixture was left to stand for 30 min in the dark. The reduction of the DPPH radical was determined by measuring the absorption at 515 nm. Radical‐scavenging activity (RSA) was calculated as a percentage of DPPH discoloration using the equation RSA (%) = [(ADPPH−AS)/ADPPH] × 100, where AS is the absorbance of the solution when the sample extract has been added at a particular level and ADPPH is the absorbance of the DPPH solution. The extract concentration providing 50% of antioxidant activity (EC_50_) was calculated from the graph of DPPH scavenging activity against extract concentrations (Sarmento et al. 2015).

Reducing power: This methodology was performed using the microplate reader described above. The different concentration solutions (0.5 mL) were mixed with sodium phosphate buffer (200 mmol/L, pH 6.6, 0.5 mL) and potassium ferricyanide (1% w/v, 0.5 mL). The mixture was incubated at 50°C for 20 min, and trichloroacetic acid (10% w/v, 0.5 mL) was added. The mixture (0.8 mL) was poured into the 48 wells, as well as deionized water (0.8 mL) and ferric chloride (0.1% w/v, 0.16 mL), and the absorbance was measured at 690 nm. The extract concentration providing 0.5 absorbance (EC_50_) was calculated from the graph of absorbance at 690 nm against extract concentrations (Sarmento et al. 2015).

TBARS inhibition: Porcine ( *Sus scrofa* ) brains were obtained from official slaughtering animals, dissected, and homogenized with a Polytron in ice‐cold Tris–HCl buffer (20 mmol/L, pH 7.4) to produce a 1:2 (w/v) brain tissue homogenate, which was centrifuged at 3000 × *g* for 10 min. An aliquot (0.1 mL) of the supernatant was incubated with the different solution concentrations (0.2 mL) in the presence of FeSO4 (10 μmol/L; 0.1 mL) and ascorbic acid (0.1 mmol/L; 0.1 mL) at 37°C for 1 h. The reaction was stopped by the addition of trichloroacetic acid (28% w/v, 0.5 mL), followed by thiobarbituric acid (TBA, 2%, w/v, 0.38 mL), and the mixture was then heated at 80°C for 20 min. After centrifugation at 3000 × g for 10 min to remove the precipitated protein, the color intensity of the malondialdehyde (MDA)–TBA complex in the supernatant was measured by its absorbance at 532 nm. The inhibition ratio (%) was calculated using the following formula: inhibition ratio (%) = [(A − B)/A] × 100%, where A and B are the absorbance of the control and the compound solution, respectively. The extract concentration providing 50% of antioxidant activity (EC_50_) was calculated from the graph of TBARS formation inhibition against extract concentrations.

Trolox served as a positive control for these assays. Results for all methods were expressed as EC_50_ or values (mg/mL), representing the extract concentration providing 50% antioxidant activity.

#### 2.5.2 Antimicrobial Activity

The antibacterial activity of the extracts was assessed using the broth microdilution method combined with the rapid p‐iodonitrotetrazolium chloride (INT) colorimetric assay (Pires et al. 2018). The extracts were tested against bacteria commonly associated with foodborne illnesses, including gram‐negative species such as *Enterobacter cloacae* (ATCC 49741), *Escherichia coli* (ATCC 25922), *Pseudomonas aeruginosa* (ATCC 9027), *Salmonella enterica* (ATCC 13076), and *Yersinia enterocolitica* (ATCC 8610), as well as gram‐positive species like *Bacillus cereus* (ATCC 11778), *Listeria monocytogenes* (ATCC 19111), and *Staphylococcus aureus* (ATCC 25923). Positive controls included streptomycin for all bacterial strains, ampicillin for all except *B. cereus* , and methicillin exclusively for *S. aureus* .

The MIC was determined by the colorimetric microbial viability based on the reduction of the INT colorant (0.2 mg/mL). Firstly, the samples were dissolved in 5% (v/v) Dimethyl sulfoxide (DMSO) and 95% of autoclaved distilled water to give a final concentration of 20 mg/mL for the stock solution. Then, 90 μL of this concentration was added to the first well (96‐well microplate) in duplicate with 100 μL of TSB. In the remaining wells, 90 μL of TSB medium was added. Afterwards, the samples were serially diluted to obtain the concentration ranges (10 to 0.03125 mg/mL). To finish, 10 μL of inoculum (standardized at 1.5 × 10^6^ Colony Forming Unit (CFU)/mL) was added to all the wells, assuring the presence of 1.5 × 10^5^ CFU. The microplates were incubated at 37°C for 24 h. The MIC of samples was detected following the addition (40 μL) of 0.2 mg/mL INT and incubation at 37°C for 30 min. MIC was defined as the lowest concentration that inhibits visible bacterial growth, determined by changing the coloration from yellow to pink if the microorganisms are viable. For the determination of MBC, 10 μL of liquid from each well that showed no change in color was plated on solid medium, Blood agar (7% sheep blood), and incubated at 37°C for 24 h. The lowest concentration that yielded no growth determines the MBC. MBC was defined as the lowest concentration required to kill bacteria. The results were reported as minimum inhibitory concentration (MIC) and minimum bactericidal concentration (MBC) and were expressed in mg per mL of the aqueous solutions of the lyophilized fruit samples.

For antifungal activity, two micromycetes were tested: *Aspergillus brasiliensis* and *Aspergillus fumigatus* . Ketoconazole was the positive control.

The micromycetes were maintained on malt agar, and the cultures were stored at 4°C and further placed in new medium and incubated at 25°C for 72 h. In order to investigate the antifungal activity, the fungal spores were washed from the surface of agar plates with sterile 0.85% saline containing 0.1% Tween 80 (v/v). The spore suspension was adjusted with sterile saline to a concentration of approximately 1.0 × 10^5^ in a final volume of 100 μL per well. The samples were first dissolved in 5% (v/v) dimethyl sulfoxide (DMSO) and 95% autoclaved distilled water to give a final concentration of 10 mg/mL for the stock solution. Afterwards, 90 μL of this concentration was added to the first well (96‐well microplate) in duplicate with 100 μL of Malt Extract Broth (MEB). The microplates were incubated for 72 h at 28°C. The lowest concentration with no visible growth (using a binocular microscope) was defined as the MIC. The minimum fungicidal concentrations (MFCs) were determined by serial sub‐cultivation of 2 μL in microtiter plates containing 100 μL of malt broth per well and further incubation for 72 h at 28°C. The lowest concentration with no visible growth was defined as the MFC, indicating 99.5% killing of the original inoculum. Results were expressed as the MIC and minimal fungicidal concentrations (MFC). The results were expressed as mg per mL of the aqueous solutions of the lyophilized fruit samples.

### 2.6 Statistical Analysis

All assays were conducted in triplicate (*n* = 3), and the results were expressed as the mean ± standard deviation (SD). Statistical analysis was performed at a 5% significance level using SPSS software (IBM SPSS Statistics for Windows, Version 23.0; IBM Corp., Armonk, NY, USA). Differences between samples were assessed through one‐way analysis of variance (ANOVA), and mean comparisons were carried out using Tukey's HSD test (*p* = 0.05). For comparisons between only two samples, a Student's *t*‐test was applied.

## 3 Results and Discussion

### 3.1 Nutritional and Chemical Characterization

The results regarding the nutritional composition of the studied *A. muricata* , *A. squamosa* , *A. senegalensis* , *A. boehmii*, *D. edulis* , and *S. spinosa* edible fruits are presented in Table 1. Carbohydrates were the predominant macronutrients found in all samples, with statistically significant differences (*p* < 0.05) detected among some. *A. muricata* exhibited the highest concentration at 93.3 g/100 g dw. At the same time, *D. edulis* contained the lowest carbohydrate content at 51.5 g/100 g dw. Proteins appeared next, with *A. senegalensis* presenting the highest concentration (15.6 g/100 g dw), followed by significant amounts detected in *S. spinosa* (8.79 g/100 g dw), *D. edulis* (7.99 g/100 g dw), *A. muricata* (7.3 g/100 g dw), and finally *A. squamosa* (5.17 g/100 g dw), which presented the lowest protein content. It is worth noting that the *Annona* species presented the most significant variability in protein content compared to the other studied fruits, which mainly suggests the potential genetic variability influencing protein accumulation in these fruits. Moreover, the considerable range in the protein content of the studied edible fruits highlights their nutritional diversity, an essential factor in countries where access to more diverse and nutritious foods is scarce. Regarding crude fat, *D. edulis* presented an exceptionally high concentration (40.5 g/100 g dw) compared to the other studied fruits. This species is renowned for its rich lipid content, which may vary from 33% to 65%. Recent studies have explored its use as a margarine substitute in different pastry products (Arisa et al. 2021; Eyenga et al. 2020), which could constitute an alternative for diversifying the sources of edible and industrial fats and making use of a nutritionally rich natural product. Relatively high fat concentrations were also found in *A. senegalensis* (6.5 g/100 g dw) compared to other fruits. This finding aligns with a study by Donhouedé et al. (2023), which reported lipid concentrations ranging from 2.52% to 4.90%, attributing these differences to variations in geographical location and growing conditions. *S. spinosa* was found to hold the highest ash concentrations (19.96 mg/100 g dw), indicative of its superior mineral richness, constituting an additional indicator of the quality of the fruits from this species.

**TABLE 1:** Nutritional value, energy content, and hydrophilic compounds in selected fruits.

	*Anonna muricata*	*Annona squamosa*	*Annona senegalensis*	*Anisophyllea boehmii*	*Dacryodes edulis*	*Strychnos spinosa*
**Nutritional value**						
Fat	2.6 ± 0.2^c^	1.4 ± 0.3^e^	6.5 ± 0.3^b^	2.2 ± 0.2^d^	40.5 ± 0.5^a^	2.2 ± 0.2^d^
Proteins	7.3 ± 0.7^e^	5.17 ± 0.5^f^	15.6 ± 0.1^a^	7.66 ± 0.3^d^	7.99 ± 0.8^c^	8.79 ± 0.9^b^
Ash	2.14 ± 0.01^f^	4.49 ± 0.06^c^	7.36 ± 0.02^b^	3.83 ± 0.03^d^	3.55 ± 0.08^e^	19.96 ± 0.07^a^
Carbohydrates	90.1 ± 0.6^b^	93.3 ± 0.1^a^	77.1 ± 0.6^c^	89.8 ± 0.4^b^	51.5 ± 0.9^d^	88.9 ± 0.4^b^
Energy	412.7 ± 0.6^cd^	411.1 ± 0.9^d^	432 ± 2^b^	412.2 ± 0.8^cd^	602 ± 2^a^	411.2 ± 0.9^c^
**Free sugars**						
Fructose	11.5 ± 0.6^b^	16.8 ± 0.1^a^	9.80 ± 0.02^c^	9.49 ± 0.04^cd^	0.86 ± 0.09^f^	8.94 ± 0.03^de^
Glucose	12.7 ± 0.7^b^	13.1 ± 0.5^a^	8.63 ± 0.09^d^	9.99 ± 0.04^c^	0.34 ± 0.07^e^	12.09 ± 0.06^b^
Sucrose	nd	1.32 ± 0.01^d^	2.00 ± 0.06^c^	18.10 ± 0.06^a^	0.07 ± 0.02^e^	6.86 ± 0.06^b^
Trehalose*	0.82 ± 0.08	nd	nd	nd	nd	0.13 ± 0.03
Total	25.01 ± 0.38^d^	31.27 ± 0.05^b^	20.43 ± 0.001^e^	37.58 ± 0.08^a^	1.27 ± 0.04^f^	28.01 ± 0.01^c^
**Organic acids**						
Oxalic	0.23 ± 0.01^c^	0.22 ± 0.01^c^	0.44 ± 0.01^a^	0.24 ± 0.01	0.37 ± 0.01^b^	0.07 ± 0.01^d^
Quinic	nd	nd	0.63 ± 0.02^b^	nd	2.69 ± 0.06^a^	0.16 ± 0.02^c^
Malic	1.87 ± 0.01^b^	0.60 ± 0.01^e^	3.25 ± 0.01^a^	0.13 ± 0.02^f^	1.66 ± 0.01^bc^	0.91 ± 0.01^d^
Total	2.10 ± 0.01^c^	0.82 ± 0.01^e^	4.32 ± 0.01b	0.37 ± 0.01^f^	4.72 ± 0.05^a^	1.13 ± 0.02^d^

*Note:* All results were expressed as mean ± SD (*n* = 3) and in g per 100 g of dried weight (dw), except for the ash content (mg/100 g/dw), and the energetic value (kcal/100 g/dw), nd‐ not detected. Different letters in the same line indicate significant differences between means according to Tukey's HSD test (*p* < 0.05) and * denotes significance (*p* < 0.001) between two samples according to the *t*‐Student test.

**FIGURE 2:**
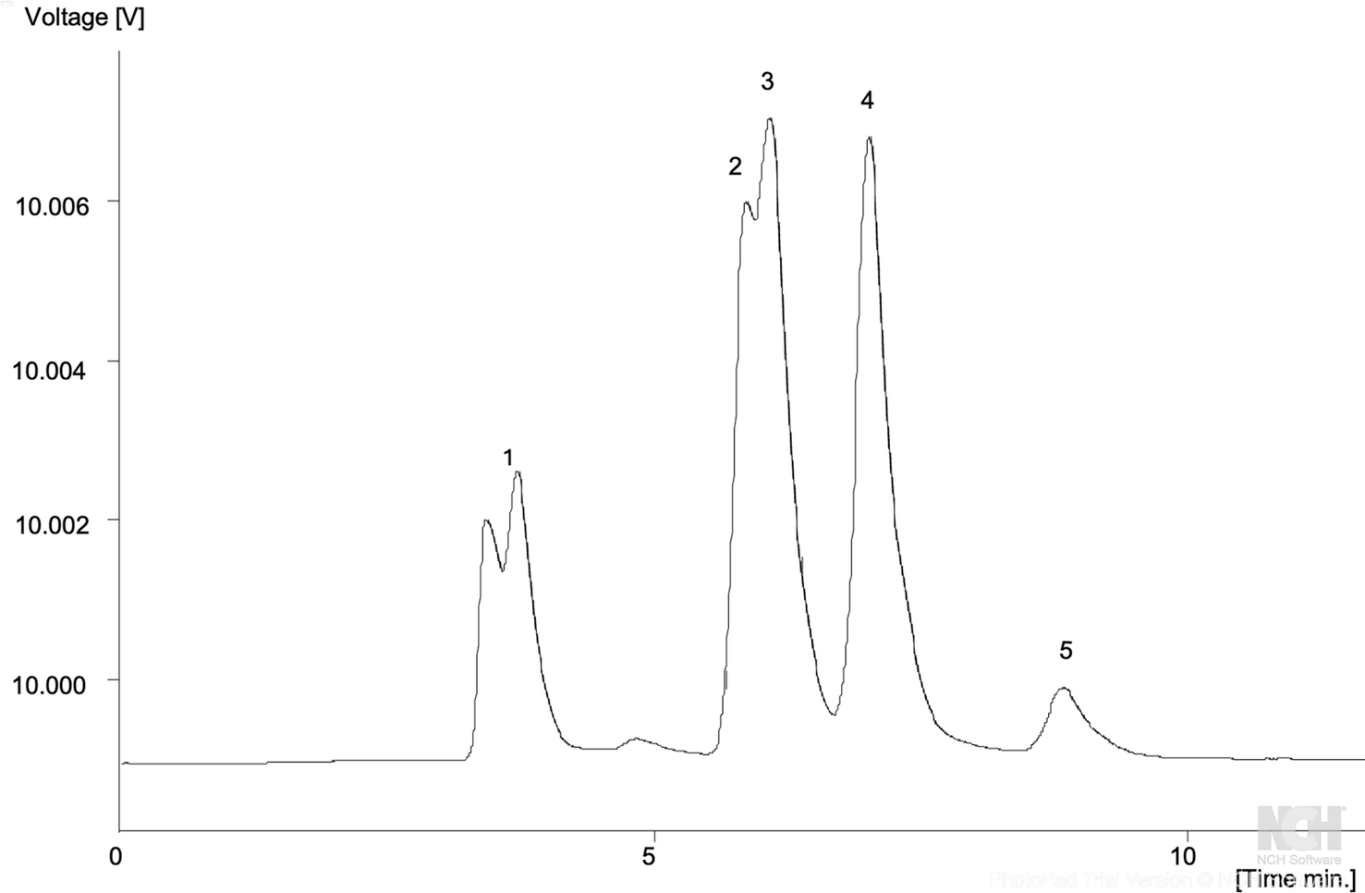
Free sugars profile of *A. boehmii* fruit. 1‐ Mobile phase, 2‐ fructose, 3‐ glucose, 4‐ sucrose, 5‐ internal standard.

Table 1 presents the free sugar profile analysis results on the studied fruits. Fructose and glucose were detected in all samples, with statistically significant differences (*p* < 0.05) among species. Notably, *A. squamosa* stood out with the highest fructose and glucose contents (16.8 and 13.1 g/100 g dw, respectively), distinguishing itself as the only species where fructose predominates. This high fructose and glucose content aligns with *A. squamosa's* reputation as one of the sweetest fruits within the *Annona* genus. Kumhar et al. (2014), who studied the sugar composition of *A. squamosa* pulp, reported 3.5% fructose, 3.4% sucrose, 5.1% glucose, and 1.2%–2.5% other oligosaccharides. These concentrations are lower than those found in our study, likely due to variations in edaphoclimatic conditions of fruit‐growing regions. Glucose, in turn, predominates in *A. muricata* (12.7 g/100 g dw), *S. spinosa* (12.09 g/100 g dw), and *A. senegalensis* (8.63 g/100 g dw), while in *A. boehmii*, sucrose was the primary sugar (18.10 g/100 g dw) (Figure 2). Previous research by Lofa et al. (2024) on *A. boehmii's* sugar content was limited to quantifying only fructose and glucose. Our study, therefore, represents the first comprehensive analysis of a broader spectrum of free sugars in this species. Among all the species studied, *D. edulis* exhibited the lowest sugar content, with only 1.27 g/100 g dw across all identified sugars. To our knowledge, this study represents the first comprehensive report on the sugar profile of *D. edulis* . The remarkably low sugar content distinguishes this species from the other fruits analyzed, potentially indicating its unique, less sweet flavor.

The analysis of the organic acids profile (Table 1) revealed the presence of three compounds, namely oxalic, quinic, and malic acids. While oxalic and malic acids were detected in all samples, quinic acid was only identified in *A. senegalensis* , *D. edulis* , and *S. spinosa* . Malic acid was the predominant organic acid quantified in all fruits, except in *A. boehmii* (0.13 g/100 g dw), where oxalic acid prevails (0.24 g/100 g dw), although also in low concentrations. Overall, while *A. boehmii* exhibited the lowest total concentration of organic acids (0.37 g/100 g dw), *D. edulis* stood out with higher levels, primarily due to the significant contributions of quinic and malic acids. Lofa et al. (2024) also studied the organic acids profile of *D. edulis* fruits from Angola, reporting the presence of citric, malic, oxalic, and ascorbic acids, with citric acid being the predominant compound (5.26 g/100 g dw), followed by also considerable concentrations of malic (0.44 g/100 g dw) and ascorbic acids (0.18 g/100 g dw). The differences between our findings and those reported by Lofa and colleagues can be attributed to possible variations in fruit maturity stages, genetic variability, and environmental factors.

The results obtained for the analysis of the fatty acid profile are shown in Table 2. A total of 13 fatty acids were identified in all samples, except in *A. boehmii*, where only 11 were identified. There were significant differences among the studied samples regarding the relative concentrations of saturated, monounsaturated, and polyunsaturated fatty acids.

**TABLE 2:** Lipophilic compounds in selected fruits.

	*Anonna muricata*	*Annona squamosa*	*Annona senegalensis*	*Anisophyllea boehmii*	*Dacryodes edulis*	*Strychnos spinosa*
**Fatty acids**						
C12:0	0.229 ± 0.001	0.47 ± 0.03	0.713 ± 0.002	2.45 ± 0.03	0.107 ± 0.004	0.183 ± 0.004
C14:0	1.449 ± 0.003	0.851 ± 0.002	0.96 ± 0.01	1.92 ± 0.03	0.247 ± 0.006	1.01 ± 0.01
C15:0	0.264 ± 0.005	0.35 ± 0.01	0.149 ± 0.001	nd	0.078 ± 0.003	0.848 ± 0.003
C16:0	29.61 ± 0.01	34.86 ± 0.01	17.85 ± 0.01	32.83 ± 0.02	58.55 ± 0.02	32.22 ± 0.06
C17:0	0.66 ± 0.05	0.77 ± 0.03	0.211 ± 0.005	nd	0.151 ± 0.001	0.46 ± 0.01
C18:0	2.11 ± 0.01	8.04 ± 0.03	3.69 ± 0.01	6.51 ± 0.01	4.84 ± 0.04	11.03 ± 0.01
C18:1n9c	26.51 ± 0.01	17.71 ± 0.01	45.15 ± 0.02	17.8 ± 0.1	24.27 ± 0.05	32.73 ± 0.01
C18:2n6c	16.06 ± 0.07	12.3 ± 0.1	21.74 ± 0.02	3.24 ± 0.03	10.82 ± 0.03	11.21 ± 0.01
C18:3n3	19.48 ± 0.01	14.82 ± 0.01	8.43 ± 0.01	7.32 ± 0.02	0.39 ± 0.01	1.03 ± 0.01
C20:0	0.52 ± 0.01	0.62 ± 0.02	0.48 ± 0.01	1.29 ± 0.01	0.183 ± 0.002	2.04 ± 0.05
C22:0	0.71 ± 0.02	1.36 ± 0.03	0.166 ± 0.003	18.07 ± 0.03	0.063 ± 0.001	2.966 ± 0.002
C23:0	1.14 ± 0.12	4.27 ± 0.06	0.065 ± 0.006	2.45 ± 0.03	0.151 ± 0.001	1.18 ± 0.02
C24:0	1.28 ± 0.03	3.54 ± 0.04	0.402 ± 0.001	6.09 ± 0.01	0.139 ± 0.002	3.16 ± 0.07
**SFA (%)**	38.0 ± 0.1^d^	55.1 ± 0.2^c^	24.68 ± 0.05^e^	71.6 ± 0.1^a^	64.51 ± 0.01^b^	55.09 ± 0.09^c^
**MUFA (%)**	26.51 ± 0.01^c^	17.71 ± 0.01^e^	45.15 ± 0.02^a^	17.8 ± 0.1^e^	24.27 ± 0.05^d^	32.73 ± 0.01^b^
**PUFA (%)**	35.54 ± 0.08^a^	27.1 ± 0.2^c^	30.17 ± 0.02^b^	10.56 ± 0.05^e^	11.21 ± 0.05^e^	12.24 ± 0.01^d^
**Tocopherols**						
α‐Tocopherol	0.62 ± 0.03^d^	0.66 ± 0.02^c^	2.29 ± 0.03^a^	1.75 ± 0.07^b^	0.32 ± 0.05e	0.27 ± 0.01^f^
β‐Tocopherol	nd	nd	nd	0.08 ± 0.02	nd	nd
γ‐Tocopherol*	nd	nd	0.81 ± 0.06	nd	0.95 ± 0.03	nd
δ‐Tocopherol	nd	nd	0.57 ± 0.08	nd	nd	nd
Total	0.62 ± 0.03^e^	0.66 ± 0.02^d^	3.68 ± 0.01^a^	1.83 ± 0.08^b^	1.27 ± 0.03c	0.27 ± 0.01^f^

*Note:* C12:0‐ lauric acid; C14:0‐ myristic acid; C16:0‐ palmitic acid; C17:0‐ heptadecanoic acid; C18:0‐ stearic acid; C18:1n9c‐ oleic acid; C18:2n6c‐ linoleic acid; C18:3n3‐ linolenic acid; C20:0‐ arachidic acid; C22:0‐ behenic acid; C23:0‐ tricosanoic acid and C24:0‐lignoceric acid; SFA‐ saturated fatty acids; MUFA‐ monounsaturated fatty acids; PUFA‐ polyunsaturated fatty acids. All results were expressed as mean ± SD (*n* = 3) and reported as relative percentage (%) for the fatty acids and in mg/100 g dw for the tocopherols, nd‐ not detected. Different letters in the same line indicate significant differences between means according to Tukey's HSD test (*p* < 0.05) and * denotes significance (*p* < 0.001) between two samples according to the *t*‐Student test.

**FIGURE 3:**
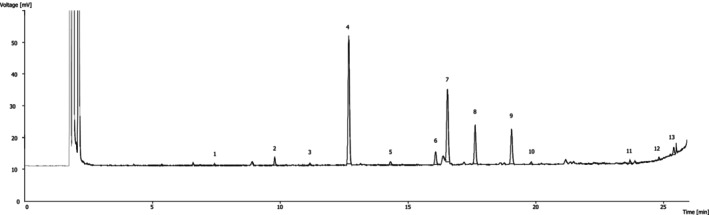
Fatty acids profile of *A. muricata* fruit. 1, C12:0; 2, C14:0; 3, C15:0; 4, C16:0; 5, C17:0; 6, C18:0; 7, C18:1n9c; 8, C18:2n6c; 9, C18:3n3; 10, C20:0; 11, C22:0; 12, C23:0; 13, C24:0.

Saturated fatty acids (SFA) were the major group identified in all of the studied fruits, except in *A. senegalensis* , where monounsaturated fatty acids (MUFA) prevailed (45.15%). The high SFA concentrations in these fruits are mainly attributed to palmitic acid (C16:0), whose concentration ranged from 29.6% to 58.5%. *A. boehmii* presented the highest SFA content (71.6%), followed by *D. edulis* (64.5%), *A. squamosa* , *S. spinosa* (55.1%), and *A. muricata* (38.0%, Figure 3). Mwagandi Chimbevo and Essuman (2019) studied the fatty acid profiles of *A. muricata* and *A. squamosa* fruits, also identifying palmitic acid as the primary component, followed by linoleic and linolenic acids. Conversely, Oluwaniyi and colleagues reported oleic acid as the predominant fatty acid in *D. edulis* fruits, which does not align with our results. In *A. muricata* , polyunsaturated fatty acids (PUFA) were identified in similar concentrations to SFA (35.5%), with linoleic (C18:2n9c) and linolenic (C18:3n3) acids as the main contributors. In *A. senegalensis* , in turn, the major concentration in MUFA is mainly triggered by the high concentration of oleic acid (45.15%). Regarding *A. boehmii*, to our knowledge, this is the first report on the fatty acid profile of the fruit, with most studies focusing on the kernel portion (Nkengurutse et al. 2016, 2019). Overall, the high SFA content detected in most studied fruits is noteworthy and may influence their dietary considerations.

Regarding tocopherol analysis (Table 2), α‐tocopherol was identified in all of the samples studied, with concentrations ranging from 0.27 to 2.29 mg/100 g dw, making it the predominant compound in all species except *D. edulis* , where γ‐tocopherol prevailed (o.95 mg/100 g dw). Additionally, γ‐ and δ‐tocopherol were detected in *A. senegalensis* , while β‐tocopherol was exclusively found in *A. boehmii* at minor concentrations (0.08 mg/100 g dw). Luzia and Jorge (2012) studied the tocopherol profile of *A. muricata* and *A. squamosa* . Contrary to our findings, they reported the presence of all four tocopherol isoforms in these species, except δ‐tocopherol in *A. squamosa* , with γ‐tocopherol being the most abundant in both species. To our knowledge, there are no previous reports on the tocopherol profile of the remaining studied fruits. Overall, tocopherol isomers, particularly α‐tocopherol, suggest potential health benefits, such as antioxidant properties.

### 3.2 Phenolic Composition

The phenolic composition of the edible fruits' hydroethanolic extracts was investigated, precisely the retention time, λ_max_, pseudomolecular ions, major fragment ions in MS^2^, and tentative identification of each compound. These were tentatively identified based on experimental data and, when possible, with existing standard compounds and published literature. The results for *A. muricata* , *A. squamosa* , and *A. senegalensis* , including identifying and quantifying individual phenolic compounds, are summarized in Table 3. A total of 22 phenolic compounds were identified, including phenolic acids and flavonoids. Among these, fifteen were identified as condensed tannins, with procyanidin derivatives standing out, accounting for nine detected compounds (**peaks 3, 10, 12, 14, 15, 16, 17, 19, and 20**). These were tentatively identified by comparison with a (+)‐catechin commercial standard. Based on their degree of polymerization, the detected compounds included procyanidin dimers (**peaks 3** and **17**; [M‐H]^−^ at *m/z* 577), trimers (**peak 19** and **20**; [M‐H]^−^ at *m/z* 865 and 831, respectively), tetramers (**peaks 10**, **12**, **14**, and **15**; [M‐H]^−^ at *m/z* 1151 (**peak 10**) and 1153), and a pentamer (**peak 16**; [M‐H]^−^ at *m/z* 1441). Moreover, both A‐type and B‐type procyanidin linkages were detected, indicating structural diversity among the identified oligomeric procyanidins. These compounds exhibited unique MS^2^ fragment ions according to the degree of polymerization. Three catechin derivatives were also tentatively identified, among them (+)‐catechin (**peak 8**; [M‐H]^−^ at *m/z* 289), epigallocatechin (**peak 11**; [M‐H]^−^ at *m/z* 305), and (−)‐epicatechin (**peak 13**; [M‐H]^−^ at *m/z* 289). Quercetin‐3‐*O*‐rutinoside (**peak 22**; [M‐H]^−^ at *m/z* 609) was tentatively identified based on its deprotonated ion and fragmentation pattern, with an MS^2^ fragment at *m/z* 301 confirming the quercetin aglycone backbone. Other identified flavonoids include eridictyol‐rutinoside (**peak 18**; [M‐H]^−^ at *m/z* 595), a flavanone linked to a sugar moiety, while lambertianin C (**peak 21**; [M‐H]^−^ at *m/z* 1401) was identified as an ellagitannin. Seven compounds were tentatively identified as phenolic acids, with coumaric acid derivatives standing out. Compounds **2, 5, 6**, and **7** were identified by comparison with commercial standards as galloyl‐*p*‐coumaric acid, 3,4‐di‐*p*‐coumaroylquinic acid, 3‐*p*‐coumarylquinic acid, and *p*‐coumaric acid hexose, respectively. Their pseudomolecular ions were detected at [M‐H]^−^ at *m/z* 315, 675, 337, and 325, respectively, with distinct daughter fragment ions indicating variations in *p*‐coumaric acid groups attached to the quinic acid backbone. Other single phenolic acids identified included a derivative of glucose oxidation (**peak 1**; [M‐H]^−^ at *m/z* 209), glutaric (**peak 4**; [M‐H]^−^ at *m/z* 131), and an isomer of chlorogenic acid, 4‐*O*‐caffeoylquinic acids (**peak 7**; [M‐H]^−^ at *m/z* 353).

**TABLE 3:** Retention time (Rt), wavelengths of maximum absorption in the visible region (λmax), mass spectral data, identification, and quantification of phenolic compounds in hydroethanolic extracts of *Annona* species fruits.

Peaks	Rt (min)	λmax (nm)	[M‐H]^−^ *m/z*	MS^2^ (*m/z*)	Tentative Identification	*A. muricata*	*A. squamosa*	*A. senegalensis*
**1**	4.31	209	324	191 (100)	Glucaric acid	0.033 ± 0.002	0.032 ± 0.003	0.077 ± 0.001
**2**	5.13	312	315	163 (100)	Galloyl‐*p*‐coumaric acid	0.011 ± 0.004	traces	0.004 ± 0.002
**3**	5.48	280	577	451 (38), 425 (67), 407 (100), 289 (76)	Procyanidin dimer isomer I	0.032 ± 0.001	0.036 ± 0.001	0.314 ± 0.007
**4**	5.61	279	131	115 (100)	Glutaric acid	0.013 ± 0.001	nd	nd
**5**	5.73	312	675	337 (100), 191 (28)	3.4‐di‐*p*‐coumaroylquinic acid	0.102 ± 0.001	0.1003 ± 0.001	0.111 ± 0.004
**6**	6.13	321	337	265 (100), 173 (21), 163 (10)	3‐*p*‐Coumaroylquinic acid	nd	0.099 ± 0.001	nd
**7**	6.73	322	353	191 (32), 179 (52) 173 (100)	4‐*O*‐caffeoylquinic acid	0.104 ± 0.001	0.1003 ± 0.001	0.185 ± 0.001
**8**	7.21	288	289	245 (100)	(+)‐Catechin	nd	nd	0.053 ± 0.001
**9**	7.69	321	325	163 (100)	*p*‐Coumaric acid hexose	0.013 ± 0.003	0.001 ± 0.001	0.011 ± 0.001
**10**	8.39	283	1151	863 (100), 575 (32), 287 (11)	A‐type Procyanidin tetramer with one A‐bond	nd	0.041 ± 0.004	nd
**11**	9.16	288	305	219 (45), 179 (41), 125 (100)	Epigallocatechin	0.039 ± 0.001	0.036 ± 0.001	0.089 ± 0.002
**12**	9.74	281	1153	1027, 2257 (35), 865, 1948 (30), 863, 1793 (65), 739, 1640 (30), 451, 1016 (45), 407, 0756 (25), 289, 0705 (100), 287, 0548 (40)	B‐type Procyanidin tetramer isomer I	nd	nd	0.201 ± 0.005
**13**	9.86	282	289	245 (100)	(−)‐Epicatechin	0.048 ± 0.002	0.038 ± 0.001	nd
**14**	10.43	281	1153	1027, 2257 (35), 865, 1948 (30), 863, 1793 (65), 739, 1640 (30), 451, 1016 (45), 407, 0756 (25), 289, 0705 (100), 287, 0548 (40)	B‐type Procyanidin tetramer isomer II	0.039 ± 0.004	nd	nd
**15**	11.64	291	1153	1027 (35), 865 (30), 863 (15), 739 (21), 451 (45), 407 (25), 289 (100)	B‐type Procyanidin tetramer isomer III	nd	0.054 ± 0.003	nd
**16**	13.85	288	1441	1153 (27), 865 (92), 577 (47), 289 (25), 287 (10)	Procyanidinn pentamer isomer I*	0.045 ± 0.001	nd	0.066 ± 0.001
**17**	14.31	288	577	451 (38), 425 (67), 407 (100), 289 (76)	Procyanidin dimer isomer II	0.035 ± 0.002	nd	nd
**18**	15.45	330	595	287 (100), 269 (31)	Eridictyol‐rutinoside	0.109 ± 0.003	0.103 ± 0.001	0.112 ± 0.001
**19**	16.14	288	865	451 (44), 425 (59), 407 (97), 289 (65)	Procyanidin trimer isomer I*	0.061 ± 0.001	nd	0.079 ± 0.003
**20**	16.34	291	831	575 (32), 287 (11)	A‐type proanthocyanidin trimer with one A‐bond	0.048 ± 0.001	nd	nd
**21**	18.38	290	1401	935 (100), 633 (38), 301 (10)	Lambertianin C	1.193 ± 0.001	nd	nd
**22**	17.91	354	609	301 (100)	Quercetin‐3‐*O*‐rutinoside	0.462 ± 0.002	0.463 ± 0.001	0.481 ± 0.005
					**Total phenolic acids**	0.279 ± 0.001^c^	0.334 ± 0.001^b^	0.388 ± 0.006^a^
					**Total flavonoids**	2.11 ± 0.01^a^	0.838 ± 0.003^c^	1.92 ± 0.02^b^
					**Total phenolic compounds**	2.39 ± 0.01^a^	1.172 ± 0.004^b^	2.3 ± 0.02^a^

*Note:* All results were expressed as mean ± SD (*n* = 3) and reported in mg/g of extract, nd‐ not detected. Different letters in the same line indicate significant differences between means according to Tukey's HSD test (*p* < 0.05) and * denotes significance (*p* < 0.001) between two samples according to the *t*‐Student test.

**FIGURE 4:**
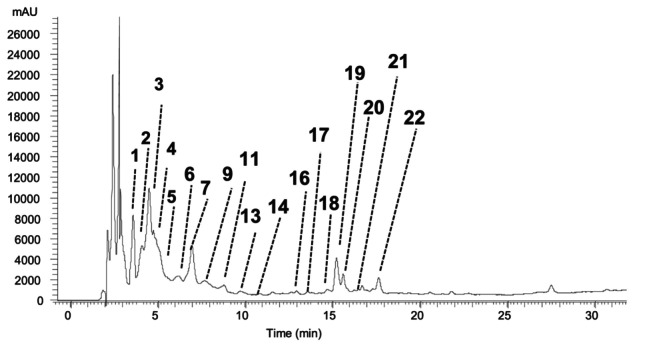
Phenolic profile of the *A. muricata* fruit hydroethanolic extracts recorded at 280 nm. Peak numbers correspond to the identification described in Table 4.

Data regarding the quantification of the phenolic compounds identified in *A. muricata* fruits' hydroethanolic extracts (Figure 4) revealed the prevalence of lambertianin C (1.193 mg/g extract). Several studies have demonstrated the biological importance of lambertianin C, an ellagitannin with proven antioxidant, anti‐inflammatory, antimicrobial, and anticancer activities (Aguilera‐Correa et al. 2021; Kashchenko et al. 2021; Lipińska et al. 2014). Quercetin‐3‐*O*‐rutinoside, or rutin, is the second most abundant compound identified in *A. muricata* (0.463 mg/g extract). This flavonoid has been broadly studied for its several health benefits, including antioxidant potential, cardiovascular protection, and neuroprotective properties (Bhat and Bhat 2021). Ochoa‐Jiménez et al. (2024) investigated the phenolic profile of *A. muricata* fruits during ripening, identifying 37 individual compounds. Consistent with our findings, their analysis revealed that procyanidin, catechin derivatives, and coumaric acid derivatives were the predominant phenolic compounds in these fruits. Additionally, they identify other significant phenolic compounds, including kaempferol derivatives, naringenin, and several other phenolic acids, with these differences potentially related to genetic and environmental factors. The diversity of phenolic compounds likely contributes to the fruit's biological activities and potential health benefits. In *A. squamosa* fruits, quercetin‐3‐*O*‐rutinoside emerged as the major compound identified, with a concentration of 0.463 mg/g extract. Notably, several compounds identified in other *Annonas* species under study, such as some procyanidin derivatives, lambertianin C, catechin, and glutaric acid, were absent in *A. squamosa* , which ultimately contributed to the fruit's relatively lower concentration of total phenolic compounds. The phenolic profile of *A. squamosa* fruits was also investigated by Baskaran and colleagues (Baskaran et al. 2016), who identified 44 free, bound, and esterified forms. These compounds were predominantly phenolic acids, including gallic, protocatechuic, synaptic, ferulic, and *p*‐coumaric. Additionally, among flavonoids, catechin and gallocatechin derivatives, caffeoylhexoside, dihydroxyquercetin, and other related compounds were also present, highlighting the complexity and diversity of the fruit's phytochemical composition. In *A. senegalensis* , although quercetin‐3‐*O*‐rutinoside was likewise the predominant phenolic compound (0.481 mg/g extract), others also stand out, namely procyanidin dimer isomer I (0.314 mg/g extract) and B‐type procyanidin tetramer isomer II (0.201 mg/g extract). Procyanidin derivatives are highly significant in biological systems due to their diverse health‐promoting properties, including antioxidant, anti‐inflammatory, and antimicrobial activities. They also play a crucial role in supporting gastrointestinal health, demonstrating anticancer potential, and contributing to various other therapeutic benefits (Valencia‐Hernandez et al. 2021). This is the first report on the phenolic composition of *A. senegalensis* fruits, providing valuable insights into their bioactive profile and potential health‐promoting properties.

Table 4 presents the tentative identification of individual phenolic compounds detected in *A. boehmii*, *D. edulis* , and *S. spinosa* . Regarding *A. boehmii* fruit hydroethanolic extract, nineteen compounds were identified, of which fifteen were flavonoids and four were phenolic acids. Among flavonoids, quercetin glycosylated prevails, with four compounds identified in this species. Quercetin‐3‐*O*‐deoxyhexoside (**peak 33**) and quercetin‐3‐*O*‐rutinoside (**peak 34**) presented the same deprotonated ion [M − H]^−^ at *m/z* 609 and a MS^2^ ion at *m/z* 301, indicative of the loss of a rutinose in both compounds. Similarly, quercetin‐3‐*O*‐hexoside (**peak 35**) and quercetin‐3‐*O*‐glucoside (**peak 36**) presented the same deprotonated ion [M − H]^−^ at *m/z* 463, presenting the same fragment of the previously described compound, revealing the loss of a hexose and a glucoside. Catechin derivatives appear next, with catechin‐di‐pentoside (**peak 4**; [M − H]^−^ at *m/z* 553), catechin‐pentoside (**peak 21**; [M − H]^−^ at *m/z* 403), and catechin‐rutinoside (**peak 32**; [M − H]^−^ at *m/z* 597) being identified in *A. boehmii* fruit extracts. In these, the fragmentation pattern suggested that the daughter ions at *m/z* 421, 289, and 383 correspond to the loss of one pentose unit (−132 Da), the catechin aglycone, and the loss of the entire rutinoside moiety (−308 Da), respectively. *O*‐methylated flavonol derivatives, namely isorhamnetin‐3‐*O*‐glucoside (**peak 18**) and isorhamnetin‐acetylhexoside (**peak 25**) were also tentatively identified, presenting deprotonated ions [M − H]^−^ at *m/z* 477 and 519, respectively, producing daughter ions concordant with the loss of the glucoside moiety (−162 Da) and the loss of the acetyl group (−86 Da). Other single flavonoids tentatively identified in *A. boehmii* included two anthocyanins, pelargonidin‐3‐*O*‐glucoside (**peak 17**; [M − H]^−^ at *m/z* 433) and cyanidin‐3‐*O*‐glucoside (**peak 23**; [M − H]^−^ at *m/z* 449), as well as myricetin‐hexoside (**peak 27**; [M − H]^−^ at *m/z* 479), a flavone conjugated with a hexose moiety. Four phenolic acids were also tentatively identified in *A. boehmii* fruit extracts, of which caffeic acid derivatives stand out, with two compounds detected (**peaks 1** and **5**). Caffeic acid‐acetyl‐di‐hexoside presented a deprotonated ion [M − H]^−^ at *m/z* 543 and daughter fragments at *m/z* 341 and 179, indicative of a loss of one hexoside unit (−162 Da) and the acetyl group (−86 Da) and the loss of both hexose units (*m/z* at 179), leaving the deprotonated caffeic acid moiety. 5‐*O*‐caffeoylquinic acid, in turn, presented a deprotonated ion [M − H]^−^ at *m/z* 353 and a single daughter fragment at *m/z* 191. Additionally, shikimic (**peak 7**; [M − H]^−^ at *m/z* 173) and coumaroylquinic (**peak 10**; [M − H]^−^ at *m/z* 337) acids were also detected in *A. boehmii* fruit extracts. Data regarding the quantification of the identified compounds revealed the prevalence of cyanidin‐3‐*O*‐glucoside as the primary anthocyanin in *A. boehmii* fruit extracts (7.47 mg/g extract), followed by also considerable amounts of quercetin (4.62–4.77 mg/g extract), myricetin (~4.62 mg/g extract), and isorhamnetin derivatives (4.61–4.66 mg/g extract). Among phenolic acids, caffeic acid derivatives prevail, with concentrations ranging from 1.45 to 1.71 mg/g extract. This is the first report on the individual phenolic compounds occurring in *A. boehmii* fruit extracts. Thus, this data provides valuable insights into the specific phenolic composition of this fruit, contributing to our understanding of its potential health benefits and biological properties.

**TABLE 4:** Retention time (Rt), wavelengths of maximum absorption in the visible region (λmax), mass spectral data, identification, and quantification (mg/g of extract) of phenolic compounds in the hydroethanolic extracts of *A. boehmii*, *S. spinosa* , and *D. edulis* fruits.

Peaks	Rt (min)	λmax (nm)	[M‐H]^−^/[M‐H]+	MS^2^ (*m/z*)	Tentative Identification	*A. boehmii*	*S. spinosa*	*D. edulis*
**1**	4.32	321	543/−	341 (100), 179 (21)	Caffeic acid‐acetyl‐di‐hexoside	1.71 ± 0.01	nd	nd
**2**	4.34	319	337/−	163 (100)	*p*‐coumaroylquinic acid*	nd	1.83 ± 0.03	2.01 ± 0.08
**3**	4.84	322	353−/	173 (100)	4‐*O‐*caffeoylquinic acid	nd	1.25 ± 0.03	nd
**4**	4.93	274	553−/	421 (30), 289 (100)	Catechin‐di‐pentoside	0.39 ± 0.04	nd	nd
**5**	5.29	321	353−/	191 (100)	5‐*O*‐caffeoylquinic acid*	1.45 ± 0.03	1.50 ± 0.03	nd
**6**	5.39	281	939−/	907 (100), 605 (21), 451 (44), 425 (8), 301 (5)	Pentagalloyl glucose	nd	nd	12.2 ± 0.06
**7**	5.51	322	173−/	127 (100)	Shikimic acid*	0.27 ± 0.04	0.38 ± 0.09	nd
**8**	5.61	268	443−/	275 (100), 143 (8)	1,3‐*O*‐Diferuloylglycerol	nd	nd	0.63 ± 0.04
**9**	5.79	276	637−/	381 (100), 269 (9), 153 (13)	Gallagic acid	nd	nd	12.01 ± 0.04
**10**	6.75	323	337−/	191 (13), 163 (100)	Coumaroylquinic acid	1.03 ± 0.02	nd	nd
**11**	7.07	298	631−/	451 (38), 301 (100)	Castalin/Vescalin	nd	nd	11.9 ± 0.02
**12**	7.31	322	191−/	173 (100)	Quinic acid	nd	0.78 ± 0.01	nd
**13**	7.38	293	613−/	445 (100), 299 (11)	Dehydrated tergallagic‐*C*‐glucoside	nd	nd	1.068 ± 0.07
**14**	7.79	288	633−/	301 (100)	Galloyl‐HHDP‐glucose (Corilagin)	nd	nd	12.39 ± 0.04
**15**	8.36	320	417−/	255 (100), 137 (21)	1,3‐*O*‐Caffeoyl‐dihydrocaffeoylglycerol	nd	1.43 ± 0.08	nd
**16**	9.53	331	341−/	299 (100), 255 (11)	Acetyl‐chrysoeriol	nd	2.22 ± 0.08	nd
**17**	10.72	520	−/433	271 (100)	Pelargonidin‐3*‐O*‐glucoside	1.79 ± 0.09	nd	nd
**18**	11.59	350	477−/	315 (100)	Ishoramnetin‐3‐*O‐*glucoside*	4.66 ± 0.01	4.73 ± 0.04	nd
**19**	11.63	326	367−/	191 (100)	Feruloylquinic acid	nd	nd	0.46 ± 0.05
**20**	11.89	524	−/449	287 (100)	Cyanidin 3‐*O*‐hexoside	4.19 ± 0.01	nd	nd
**21**	12.78	274	403−/	289 (100)	Catechin‐pentoside	0.67 ± 0.01	nd	nd
**22**	13.38	288	303−/	271 (100)	Methoxy‐naringenin	nd	1.03 ± 0.09	nd
**23**	13.56	525	−/449	287 (100)	Cyanidin 3‐*O*‐glucoside	7.47 ± 0.02	nd	nd
**24**	14.81	350	625−/	317 (100)	Myricetin‐rutinoside	4.62 ± 0.01	nd	nd
**25**	15.06	351	519−/	433 (100), 315 (25)	Ishoramnetin‐acetylhexoside	4.61 ± 0.01	nd	nd
**26**	15.37	520	−/403	271 (100)	Pelargonidin‐3‐*O*‐pentoside	1.81 ± 0.04	nd	nd
**27**	15.54	350	479−/	317 (100)	Myricetin‐hexoside*	4.61 ± 0.01	4.78 ± 0.09	nd
**28**	15.61	299	469−/	441 (100)	Valoneic acid dilactone	nd	nd	12.07 ± 0.03
**29**	16.61	325	381−/	179 (21), 161 (100), 135 (35)	1,3‐*O*‐Dicoumaroylglycerol	nd	0.05 ± 0.03	nd
**30**	16.64	290	783−/	633 (100), 615 (61), 483 (31), 313 (9), 301 (13)	Pedunculagin (bis‐HHDP‐glucose)	nd	nd	12.08 ± 0.05
**31**	17.01	355	593−/	447 (100), 301 (35)	Quercetin‐di‐rhamnoside	nd	nd	4.60 ± 0.02
**32**	17.44	285	597−/	383 (12), 289 (100)	Catechin‐rutinoside	1.28 ± 0.06	nd	nd
**33**	17.65	353	609−/	301 (100)	Quercetin‐3‐*O*‐deoxyhexoside	4.62 ± 0.01	nd	nd
**34**	17.84	354	609−/	301 (100)	Quercetin‐3‐*O*‐rutinoside	4.77 ± 0.07	nd	nd
**35**	18.75	350	463−/	301 (100)	Quercetin‐3‐*O*‐hexoside*	4.63 ± 0.05	nd	4.61 ± 0.01
**36**	19.14	357	463−/	301 (100)	Quercetin‐3‐*O*‐glucoside*	4.66 ± 0.02	nd	4.66 ± 0.08
**37**	19.61	327	381−/	179 (100)	1,4‐*O*‐Dicoumaroylglycerol	nd	0.24 ± 0.05	nd
**38**	21.59	341	447−/	285 (100)	Kaempferol‐hexoside	nd	nd	4.61 ± 0.1
**39**	22.81	347	447−/	301 (100)	Quercetin‐3‐O‐rhamnoside	nd	nd	5.03 ± 0.02
**40**	28.29	315	1267−/	929 (9), 483 (21), 301 (100)	diHHDP‐di‐galloyl‐di‐glucose	nd	nd	11.90 ± 0.01
**41**	30.25	352	477−/	315 (100)	Isorhamnetin‐3‐*O*‐glucoside	nd	nd	4.63 ± 0.02
**42**	33.98	353	461−/	315 (100)	Isorhamnetin‐3‐*O*‐rhamnoside	nd	nd	4.65 ± 0.04
					**Total anthocyanin**	15.28 ± 0.03	nd	nd
					**Total phenolic acids**	4.47 ± 0.04^c^	7.5 ± 0.1^b^	88.9 ± 0.1^a^
					**Total flavonoids**	39.57 ± 0.04^a^	12.8 ± 0.1^c^	32.82 ± 0.02^b^
					**Total compounds**	59.3 ± 0.1^b^	20.3 ± 0.2^c^	121.7 ± 0.1^a^

*Note:* All results were expressed as mean ± SD (*n* = 3) and reported in mg/g of extract, nd‐ not detected. Different letters in the same line indicate significant differences between means according to Tukey's HSD test (*p* < 0.05) and * denotes significance (*p* < 0.001) between two samples according to the *t*‐Student test.

In *S. spinosa* , twelve phenolic compounds were tentatively identified, of which seven were flavonoids and five phenolic acids. As in *A. boehmii*, isorhamnetin‐3‐*O*‐glucoside (**peak 18**) and myricetin‐hexoside (**peak 27**) were identified, these being the major compounds found in *S. spinosa* fruit extracts (4.73 and 4.78 mg/g extract). 1,3‐*O*‐caffeoyl‐dihydrocaffeoylglycerol (**peak 15**; [M − H]^−^ at *m/z* 417) and acetyl‐chrysoeriol (**peak 16**; [M − H]^−^ at *m/z* 341) come next among the most prevalent flavonoids (1.43 and 2.22 mg/g extract). Acetyl‐chrysoeriol is a derivative of chrysoeriol, a flavonoid with proven biological relevance, namely regarding its antioxidant, anti‐inflammatory, anticancer, and neuroprotective potential (Aboulaghras et al. 2022). Methoxy‐naringenin (**peak 22;** [M − H]^−^ at *m/z* 303) and 1,3‐*O*‐dicoumaroylglycerol (**peak 29;** [M − H]^−^ at *m/z* 381) were also identified, although in lower amounts (1.03 and 0.05 mg/g extract). As concerning phenolic acids, caffeic acid derivatives prevail, with 4‐*O*‐caffeoylquinic (**peak 3**; [M − H]^−^ at *m/z* 353) and 5‐*O*‐caffeoylquinic (**peak 5**) acids being identified at 1.25 and 1.50 mg/g extract. *p*‐Coumaroylquinic acid was the major phenolic acid found in *S. spinosa* fruit extract (1.83 mg/g extract), along with minor concentrations of shikimic (**peak 7**; [M − H]^−^ at *m/z* 173) and quinic (**peak 11**; [M − H]^−^ at *m/z* 631) acids. This is also the first report on individual phenolic compounds in *S. spinosa* fruit extracts, which further reinforces the importance of the presented data on the overall knowledge of this biologically relevant edible fruit.

Regarding *D. edulis* , eighteen phenolic compounds were detected, among them fourteen flavonoids and four phenolic acids. Among flavonoids, quercetin derivatives prevail, with four compounds tentatively identified, namely quercetin‐3‐*O*‐hexoside (**peak 35**) and quercetin‐3‐*O*‐glucoside (**peak 36**), both also detected in *A. boehmii*, quercetin‐*di*‐rhamnoside (**peak 31**; [M − H]^−^ at *m/z* 597), and quercetin‐3‐*O*‐rhamnoside (**peak 39**; [M − H] − at *m/z* 447). Isorhamnetin derivatives follow, with isorhamnetin‐3‐*O*‐glucoside (**peak 41**; [M − H]^−^ at *m/z* 447) and isorhamnetin‐3‐*O*‐rhamnoside (**peak 42**; [M − H]^−^ at *m/z* 461) detected in similar concentrations (4.63 and 4.65 mg/g extract). With respect to hydrolysable tannins, galloyl‐HHDP‐glucose (corilagin), in turn, was the major compound identified in *D. edulis* fruit extracts (12.39 mg/g extract), which presented a deprotonated [M − H]^−^ at *m/z* 633, followed by pentagalloyl glucose (**peak 6;** [M − H]^−^ at *m/z* 939), castalin (**peak 11**; [M − H]^−^ at *m/z* 631), pedunculagin (bis‐HHDP‐glucose) (**peak 30**; [M − H]^−^ at *m/z* 783), and diHHDP‐di‐galloyl‐di‐glucose **peak 40**; pedunculagin (bis‐HHDP‐glucose) (**peak 30**; [M − H]^−^ at *m/z* 1267). The concentrations of these hydrolysable tannins ranged from 11.9 to 12.2 mg/g extract. Corilagin has been studied for its high biological relevance, namely regarding its antioxidant and anti‐inflammatory properties (Li et al. 2018), anticancer (Gupta et al. 2019), and hepatoprotective effects (Liao et al. 2022), among others, which highlight its potential as a natural therapeutic agent for various health conditions. Other minor compounds included 1,3‐*O*‐diferuloylglycerol (**peak 30**; [M − H] − at *m/z* 443), dehydrated tergallagic‐*C*‐glucoside (**peak 13**; [M − H]^−^ at *m/z* 613), and kaempferol‐hexoside (**peak 38**; [M − H]^−^ at *m/z* 447). Among phenolic acids, valoneic acid dilactone (**peak 28**; [M − H]^−^ at *m/z* 469) and gallagic acid (**peak 9**; [M − H]^−^ at *m/z* 637) prevail (12.07 and 12.01 mg/g extract), followed by lower concentrations of *p*‐coumaroylquinic and feruloylquinic acids (**peak 2** and **19**, respectively). Atwodi and colleagues (2009) studied the profile of individual phenolic compounds occurring in *D. edulis* fruit extracts, identifying only six compounds, among them two quercetin and gallate derivatives, catechol, and ellagic acid. To our knowledge, no further studies have been published in the literature regarding the overall phenolic composition of *D. edulis* fruit extracts.

### 3.3 Bioactive Properties

#### 3.3.1 Antioxidant Activity

The antioxidant activity results for hydroethanolic extracts of *A. muricata* , *A. squamosa* , *A. senegalensis* , *A. boehmii*, *D. edulis* , and *S. spinosa* fruits are summarized in Table 5. Three different assays were employed to evaluate the antioxidant capacity of the extracts, each assessing distinct parameters of antioxidant activity. For *A. muricata* , the highest antioxidant activity was observed in the TBARS assay, with an EC_50_ value of 42 μg/mL, indicating strong lipid peroxidation inhibition. This was complemented by notable performance in the reducing power assay (EC_50_ = 85 μg/mL), showcasing its ability to donate electrons. Regarding *A. squamosa* , *A. boehmii*, and *S. spinosa* , the highest antioxidant capacity values were also accomplished in the TBARS assay, with EC_50_ values ranging from 33 to 40 μg/mL. Notably, *D. edulis* and *A. senegalensis* exhibited the strongest antioxidant activity, evidenced by the lowest EC_50_ values across assays. These results suggest that both fruits have potent antioxidant activity, making them promising sources of natural antioxidants. The stronger antioxidant capacity of the studied fruit extracts is supported by their rich profile in individual phenolic compounds (Tables 3 and 4), which include significant concentrations of gallic acid, p‐coumaric acid, ferulic acid, among others, all of which are known for their potent antioxidant capacity. These results align with others confirming the studied fruits' antioxidant performance (Lofa et al. 2024; Md Roduan et al. 2019; Moffo Foning et al. 2022; Shehata et al. 2021).

**TABLE 5:** Antioxidant activity of hydroethanolic extracts from selected fruits.

	DPPH	Reducing power	TBARS
*A. muricata*	250 ± 23^a^	85 ± 4^b^	42 ± 5^c^
*A. squamosa*	219 ± 11^b^	557 ± 17^a^	35 ± 4^c^
*A. senegalensis*	0.25 ± 0.02^c^	11.7 ± 0.2^b^	176 ± 26^a^
*A. boehmii*	321 ± 11^b^	793 ± 41^a^	40 ± 3^c^
*D. edulis*	6.9 ± 0.5^b^	71 ± 1^a^	0.18 ± 0.01^c^
*S. spinosa*	423 ± 8^b^	773 ± 34^a^	33 ± 2^c^

*Note:* All results were expressed as mean ± SD (*n* = 3) and reported as EC_50_ (μg/mL) which represents the extract concentration corresponding to 50% antioxidant activity or 0.5 absorbance for the reducing power assay; nd‐ not detected. Different letters in the same line indicate significant differences between means according to Tukey's HSD test (*p* < 0.05). Positive controls trolox EC_50_ values: 43 ± 2 μg/mL (DPPH), 30 ± 1 μg/mL (reducing power), and 21.8 ± 0.2 μg/mL (TBARS).

#### 3.3.2 Antimicrobial Activity

The antimicrobial activity results of the hydroethanolic extracts from the studied fruits are summarized in Table 6. For *A. muricata* , the highest antibacterial activity was observed against *S. enterica* and *L. monocytogenes* , with a MIC value of 1.25 mg/mL. *D. edulis* and *S. spinosa* extracts also demonstrated comparable activity against *B. cereus* and *Y. enterocolitica* , representing their best performances. *A. squamosa* exhibited the strongest antimicrobial activity among the tested extracts, with a MIC value of 0.3 mg/mL against *Y. enterocolitica* , outperforming all other extracts and bacterial cultures. Similarly, *A. senegalensis* and *A. boehmii* displayed notable antimicrobial activity, with MIC values of 0.6 mg/mL against *S. aureus* and *Y. enterocolitica* , indicating their potential as effective antibacterial agents.

**TABLE 6:** Antimicrobial activity in hydroethanolic extracts of selected fruits.

	*A. muricata*	*A. squamosa*	*A. senegalensis*	*A. boehmii*	*D. edulis*	*S. spinosa*	Positive Controls		
Antibacterial activity							Streptomicin	Methicilin	Ampicillin
	MIC/MBC	MIC/MBC	MIC/MBC	MIC/MBC	MIC/MBC	MIC/MBC	MIC/MBC	MIC/MBC	MIC/MBC
*E. cloacae*	5/> 10	10/> 10	> 10/> 10	5/> 10	10/> 10	10/> 10	0.007/0.007	nt/nt	0.15/0.15
*E. coli*	10/> 10	10/> 10	10/> 10	5/> 10	10/> 10	5/> 10	0.01/0.01	nt/nt	0.15/0.15
*P. aeruginosa*	2.5/> 10	10/> 10	> 10/> 10	> 10/> 10	10/> 10	> 10/> 10	0.06/0.06	nt/nt	0.63/0.63
*S. enterica*	1.25/> 10	2.5/> 10	5/> 10	2.5/> 10	5/> 10	2.5/> 10	0.007/0.007	nt/nt	0.15/0.15
*Y. enterocolitica*	2.5/10	0.3/> 10	1.25/> 10	0.6/10	5/> 10	1.25/> 10	0.007/0.007	nt/nt	0.15/0.15
*B. cereus*	2.5/> 10	> 10/> 10	1.25/> 10	10/> 10	1.25/> 10	10/> 10	0.007/0.007	nt/nt	n.t/n.t
*L. monocytogenes*	1.25/> 10	10/> 10	2.5/> 10	5/> 10	10/> 10	2.5/> 10	0.007/0.007	nt/nt	0.15/0.15
*S. aureus*	5/> 10	10/> 10	0.6/> 10	10/> 10	5/> 10	10/> 10	0.007/0.007	0.007/0.007	0.15/0.15

Antifungal activity							Ketoconazole
	MIC/MFC	MIC/MFC	MIC/MFC	MIC/MFC	MIC/MFC	MIC/MFC	
*A. brasiliensis*	> 10/> 10	> 10/> 10	5/> 10	10/> 10	10/> 10	10/> 10	0.06/0.125
*A. fumigatus*	10/> 10	10/> 10	5/> 10	> 10/> 10	10/> 10	10/> 10	0.5/1

*Note:* All results were expressed in mg/mL. Abbreviations: MBC, minimal bactericidal concentration; MFC, minimum fungal concentrations; MIC, minimal inhibitory concentration; nt, not tested.

These findings underscore the potential of fruit extracts, mainly from *A. squamosa* , *A. senegalensis* , and *A. boehmii*, as natural antimicrobial agents with promising applications in food preservation and safety. Regarding antifungal activity, only *A. senegalensis* demonstrated effectiveness against both *A. brasiliensis* and *A. fumigatus* , with MIC values of 5 mg/mL. The antibacterial capacity of the extracts is linked to their rich composition of individual phenolic compounds, consistent with findings from previous studies (da Trindade et al. 2020; Prince Lekpoabari et al. 2021). To our knowledge, this is the first report on the antimicrobial capacity of *A. senegalensis* , *A. squamosa* , and *S. spinosa* fruit extracts.

In summary, the nutritional and biochemical content identified in the selected fruits plays a significant role in addressing the pressing nutritional challenges in the region. Many of these fruits, such as *A. muricata* and *D. edulis* , are rich in vitamins and bioactive compounds that are crucial for human health. Given their high antioxidant content, these fruits can contribute to improving dietary diversity and addressing common deficiencies in key nutrients (Zubaidi et al. 2023).

In regions with limited access to a diverse range of foods, particularly in rural and underserved areas, incorporating such fruits into local diets could provide a sustainable solution to combat malnutrition and food insecurity. Furthermore, these fruits are often adapted to local growing conditions, making them a viable option for cultivation and consumption in the face of climate change and agricultural challenges. By promoting their cultivation and consumption, we can enhance nutritional security, improve public health, and support biodiversity conservation (Knez et al. 2023).

Therefore, the study of these native fruits is not only important from a scientific perspective but also has practical implications for improving the nutritional health of communities in Angola and similar regions facing nutritional challenges (Mgwenya et al. 2025).

## 4 Conclusion

This study highlights the nutritional and bioactive potential of *A. muricata* , *A. squamosa* , *A. senegalensis* , *A. boehmii*, *D. edulis* , and *S. spinosa* as valuable sources of bioactive compounds and nutrients. The fruits demonstrated notable variability in macronutrient content, with *A. muricata* showing the highest carbohydrate concentration and *D. edulis standing* out for its fat content. Phenolic compounds, including flavonoids and phenolic acids, were abundant, with distinct profiles across species, contributing to strong antioxidant and antimicrobial properties, particularly in *D. edulis* and *A. senegalensis* .

These findings provide a foundation for exploring the fruits' applications in food, nutraceutical, and pharmaceutical industries, emphasizing their potential to improve health, especially in regions with limited access to diverse nutritional sources. Future research should focus on deeper pharmacological investigations of these fruits' bioactive compounds, including their mechanisms of action. Additionally, studies on the scalability of cultivation, sustainable harvesting methods, and the economic potential of these fruits could foster their integration into local markets. Further exploration of their synergistic interactions with other food sources, as well as their role in tackling food security challenges, will also be crucial for their broader adoption.

## Author Contributions

**Josefa Rangel:** formal analysis (equal), investigation (equal), writing – review and editing (equal). **Ângela Liberal:** investigation (equal), writing – original draft (lead). **Tânia C. S. P. Pires:** formal analysis (equal), writing – review and editing (equal). **Tiane C. Finimundy:** formal analysis (equal), writing – review and editing (equal). **Lillian Barros:** funding acquisition (lead), project administration (equal), visualization (equal), writing – review and editing (equal). **Filipa Monteiro:** writing – review and editing (equal). **Maria M. Romeiras:** conceptualization (equal), supervision (equal), validation (equal), writing – review and editing (equal). **Ângela Fernandes:** conceptualization (lead), supervision (lead), visualization (lead), writing – review and editing (lead).

## Ethics Statement

The authors have nothing to report.

## Consent

The authors have nothing to report.

## Conflicts of Interest

The authors declare no conflicts of interest.

## Data Availability

The data that support the findings of this study are available on request from the corresponding author.
